# Plasticity and Awareness of Bodily Distortion

**DOI:** 10.1155/2016/9834340

**Published:** 2016-08-18

**Authors:** Mariella Pazzaglia, Marta Zantedeschi

**Affiliations:** ^1^Department of Psychology, University of Rome “La Sapienza,” Via dei Marsi 78, 00185 Rome, Italy; ^2^IRCCS Santa Lucia Foundation, Via Ardeatina 306, 00179 Rome, Italy

## Abstract

Knowledge of the body is filtered by perceptual information, recalibrated through predominantly innate stored information, and neurally mediated by direct sensory motor information. Despite multiple sources, the immediate prediction, construction, and evaluation of one's body are distorted. The origins of such distortions are unclear. In this review, we consider three possible sources of awareness that inform body distortion. First, the precision in the body metric may be based on the sight and positioning sense of a particular body segment. This view provides information on the dual nature of body representation, the reliability of a conscious body image, and implicit alterations in the metrics and positional correspondence of body parts. Second, body awareness may reflect an innate organizational experience of unity and continuity in the brain, with no strong isomorphism to body morphology. Third, body awareness may be based on efferent/afferent neural signals, suggesting that major body distortions may result from changes in neural sensorimotor experiences. All these views can be supported empirically, suggesting that body awareness is synthesized from multimodal integration and the temporal constancy of multiple body representations. For each of these views, we briefly discuss abnormalities and therapeutic strategies for correcting the bodily distortions in various clinical disorders.

## 1. Introduction

When considering the issue of distorted body representations, it is important to first clarify the relevant terms. The “body” is a complex physical object between the world and the brain, and the term “body representation” refers to the immediate prediction, construction, and evaluation of one's own corporal structure and space and those of other bodies. This account will not address the controversies around different definitions of the body (body schema, body image, body ideal, body model, body semantics, body structure, and topological body), which are variously invoked to explain clinical “disturbances” following brain damage. As defined here, body representation refers to the systematic, but temporary, maps that encode inner and outer sensations, positions, extensions, and anthropometric aspects of the body and form the basis of the corporal structure and immediate functioning in real time. It does not refer to the affective, aesthetic, and idealized aspects implicit in the everyday use of the term. Here, we review body representation and bodily distortion within the context of plasticity in general and awareness in particular. We do this by considering contributions from three different sources, while acknowledging that these may sometimes overlap or be contradictory. First, one may rely on an awareness of the body that is based mainly on information about vision and location. This predicts body image adjustments that are specific to the ways in which the body appears but with no strong correspondence to implicit body morphology. Second, the mental representation of the body may reflect an innate organization within the brain areas that represent the predictable and constant body, preserving bodily integrity and continuity amid ongoing change. Third, and more accurately, body image may be based on neural efferent (motor) and afferent (sensory) information. This predicts major distortions in the neural representation of the body as a result of changes in immediate sensorimotor experiences. All three systems of knowledge have some empirical support, suggesting that body awareness and alterations thereof are, in fact, synthesis of multiple sources of information and their multisensory integration. For each of these contributing systems, we also briefly discuss therapeutic strategies to correct one or more bodily distortions in people with a diseased body and an intact brain, such as those with spinal cord injuries (SCI).

## 2. Visual Input in the Construction of Body Distortions

How the human brain mediates the experience of the body has generally been examined and guided from the perspective of disturbances to bodily representations following brain damage [1–3]. In healthy conditions, ordinary inner and outer experiences of the body have been evaluated as highly reliable, precise, and even immune to error [4]. In the Body Image Task [5], for example, noninjured persons are able to accurately select templates matching their body size and to correctly produce tiles depicting isolated body parts. More recently, however, a quantitative, implicit alternative to the body map has been used in participants with an intact brain, demonstrating distortions in the representations of the body metric of healthy subjects [6]. Participants identify the location of different body parts on a touch screen, relative to the anchor (head, foot). Veridical body dimensions were compared to the relative judged locations of each body landmark to construct a perceptual body map. This metric, which is an implicit adaptation of the Body Image Task, revealed a common overestimation of body width relative to height [6], suggesting that regular distortions are related to position and shape between body regions. Additionally, in building a systematic map of the complete body, the measurement of individual body parts may be a logical prerequisite. Distorted measures of width/height are documented, not only for the complete body, but also for its parts—even those that are well represented, such as the hand [7]. Neurally, although the right parietal areas [8] and insula play a crucial role in processing the metric components of body representation, recent studies have emphasized the importance of a large set of visual areas [9, 10]. The lateral occipitotemporal region is implicated in the visual processing of the unified body and its individual parts, and these areas are not active when viewing noncorporeal objects [10, 11]. Among those with eating disorders, the implicit distortion of the body's dimensions maintains inappropriate attitudes to body shape and weight; visually, sufferers perceive themselves to be normal or fat, even if they are emaciated [12]. In such individuals, neuroimaging studies have demonstrated more marked functional and structural alterations in these visual areas, with anomalies in body image processing related to the self but not to others [13, 14]. A specific association between therapy-related changes and normalization of the BOLD signal in these areas suggests that this type of distortion of the body's configuration is based on visual contributions [15].

Additionally, body shape and size differ in terms of the presence of cross-modal illusory information showing the body stretching and shrinking, indicating that the brain “believes” the visual information and gives it precedence over proprioceptive and tactile cues [16, 17]. When multisensory information is simultaneously available, healthy humans display a robust tendency to rely on visual information rather than other forms of sensory modalities, particularly when dealing with spatial and metric tasks [18–20]. However, the distortion of the body, even when apparently driven by vision, may be modulated by multimodality [18, 21]. In the absence of vision, the distance between two touches on the body in healthy subjects is perceived to be greater when the two points run across rather than along the body, indicating that the distortion is not only visual [22, 23]. Additionally, following anaesthesia, healthy individuals continue to experience the size, shape, and posture of their body as usual, but there are also more pronounced distortions of their perceived body size [24]. The transient effect of an anaesthetic increases the distortion of the perceived body size, suggesting that body distortions could be the outcome, enhanced when these are not filtered through somatic and proprioceptive peripheral signals [24] or more generally processed multisensorily. In case of anaesthesia the distortions occur without visual information being consulted, in a way that resembles what happens to healthy subjects in the adaptation of the Body Image Task previously described. What provokes the disperception is not seeing the anaesthetized body region, for instance, but the silencing of some receptive cells that is followed by a shift of the multimodal inputs to the body neural representation. In some ways, individuals in absence of sensory motor signals from peripheral body parts may also paradoxically be more sensitive to visual body configurations and possible distortions per se. In this regard, the subjective broadening of the shoulders in insentient and immobile patients with an SCI is interesting and reflects the referencing of body width to the width of a wheelchair [25–27], suggesting that the absence of normal body sensations may play a larger role in the perceptual recalibration of the body [28, 29]. When using salient body-assistive tools, the persistent visual motor exposure to an assistive device becomes an important mediator of implicit corporeal knowledge. Expert tool use alters aspects of body representation in both nonhuman primates [30] and human adults [31] and is essential to the injured body in those such as immobile and insentient patients with SCI [32, 33]. The functional normalization of the body with tools prompts feelings of enhanced awareness by way of constant and vigilant visual guidance of the calibration of an insentient body [29, 34], with inevitable implications for the new bodily perspective of altered images of the “body-plus-wheelchair” [35]. However, distorted body configuration in patients with SCI is independent of the body part considered; overestimations of width compared to height occur for different body parts, such as the shoulder and ankle, independent of the degree of sensory motor interruption, suggesting a plastic experience of body shape that is broadly mediated by visual means, while also involving tactile and postural signals [36]. Although the eyes create what we call an image of the body, the implicit representation of the body typically occurs within a multimodal context and is remarkably malleable. Accordingly, evidence of a distorted, implicit image of the body that we consider to be visual is, in fact, also tactile and proprioceptive.

As there are no receptors with which to capture body metrics, the adjustments in perceived body size seem to have formed indirectly in the brain, marking how the body appears when regularly updated [37]. While multisensory flows provide further information about the implicit body map and cause its distortion, the stability of our experience of the body is filtered through a predominantly innate representation thereof and can be massively altered in response to sensory motor experiences.

## 3. An Innate Body Map Updated in the Human Brain

Throughout life, body size and morphology change flexibly across short time-scales. As individuals pass through different stages of development, their health, nutrition, physical activity, tool use, and other factors can cause modifications to the size and shape of the body, with adaptations occurring slowly across the life-span.

Perceptual information about body morphology is correctly computed and scaled in relation to changes in body size, such that, from infancy [38] to old age [39], a human can calculate the spatial requirements needed to allow their body to pass through a confined region. Even pregnant women, despite rapid changes to their body form, will flexibly and specifically adapt to changes in their body configuration, readily scaling their perceptions to their actual body dimensions to enable them to fit and move through openings of various sizes [40]. Novice wheelchair users, on the other hand, fail to fully recalibrate to the spatial requirements of a wheelchair when judging the body-wheel dimensions required to fit through an opening [41], suggesting that adaptation does not occur rapidly and distinguishes between the self and nonself. However, over a longer time-scale, many wheelchair users and/or people with SCI perceive their transformed body's edges as including their assistive device [29]. These results suggest that, beyond the genetically prewired body representation, body mapping remains an inherently plastic process that is formed and constantly modified by an individual's experiences [42]. The brain acquires a realistic and accurate perception of the body in part through a flow of immediate multisensory information and in part from the temporal constancy of a stored body image, providing a unique representation of the body that enables it to move appropriately in the world. Nevertheless, most neuropsychological theories (e.g., Melzack's neuromatrix hypothesis [43] and Ramachandran's reorganization hypothesis [44]) agree that the brain maintains a relatively persistent representation of one's own body. The hard-wired signature of the body is apparent from the second year of life [45]. According to the genetically determined view, this time-frame may reflect the innate organization incorporated in the sensorimotor cortices [43]. This hypothesis suggests that the phantom limb phenomenon (in which patients experience the vivid sensation that a missing limb remains attached to the body and is in synchrony with other body parts [44]) emerges in amputees and patients with congenital amelia (congenital absent limb) because the neuromatrix spontaneously generates in the brain a corporeal experience of bodily unity, continuity, and constancy, even in the absence of inputs from a missing or deafferented limb. Approximately 60–95% of these individuals experience phantom sensations of their amputated limb [43, 46], suggesting that an imprinted representation of the body is built into the brain's topography. The functionally closely linked somatosensory and motor cortices maintain this distorted, somatotopic representation of the body and are part of the discriminative pain network. A temporary [47] or permanent [48] blockage in the peripheral body region may contribute to the experience of a phantom limb, indicating the immediate need to consciously construct the body as it usually appears. Although generally following somatotopic mapping, plastic changes may not always match orderly the proximal spatial relationships in the human brain [49, 50], indicating that referred bodies may exhibit no strong isomorphism with respect to the morphology of the physical or the neural body. Sometimes, the reawakening of a previously disappeared body part may be disturbing, as in the case of phantom pain. Rather than being just an abnormal perception, the link between body incongruence and the neural plasticity resulting from sensory deprivation causes some pain states. Hence, it is possible that the pain state preserves local functional and structural representations of the missing body, driving the plastic changes after deafferentation [51]. According to this view, nonpainful sensations would be a less prominent marker of somatosensory plasticity [52], although different studies have shown that the functional reorganization of the body is not related to painful phenomena. Consequently, the dissociation, instead of the association, between pain and coexisting body distortions would be more informative. Patients with pain describe their deafferented limb as heavy, floating, swollen, and more enlarged than it really is, or they maintain that its position is different to its actual position, indicating that the painful sensation of the affected limb changes dynamically in reference to body distortions [53]. However, other studies have demonstrated that conscious distortions of the body also exist in the deafferented body, along with functional reorganization, even in the absence of phantom limb pain [53]. Importantly, homogenous or distinct plastic changes can occur among the different areas comprising the somatosensory cortex, each containing a separate body representation [54] with nociceptive and somatic abnormalities. This picture may help to reconcile the discrepancy between reports of the predominant pain, or no pain, involved in body distortions and neuroplasticity, although future studies are needed to confirm this.

Therapeutic approaches have been thought to be important for acting on the consciously felt, more stable, image of body also improving the pain. So, in a deafferented body, the use of visual and somatic inputs to normalize the body image (imaginative limb stretching, healthy limb to mirror, myoelectric prosthesis) [44], as well as pharmacological treatments that produce a reduction in the cortical reorganization of the body [55], have an analgesic effect. Substituting a more vague impression of the body may be fundamental to the body's functional proprieties or may simply confer greater responsivity and bodily awareness [42].

In both healthy and deafferented individuals, multisensory experimental stimulation can trigger the complete representation of one's own body. Despite the absence of a limb and any tactile or proprioceptive processing, visual-tactile interventions seem to elicit the reemergence of a coherent mental representation of one's own insentient body, for instance, during the rubber hand illusion (RHI) in individuals with SCI [28, 56, 57]. We know that in these individuals the illusory ownership during the RHI does not occur when stimulation is applied to numb body parts; it is only through residual sensations that the synchronous stimulation of the sentient dermatomes induces the sensation of body completeness and can reawaken body awareness [28, 56]. The illusory attribution of a rubber hand produces not only a temporarily induced analgesic effect, but also a progressive and spreading normalization of tactile sensations. Whether spontaneous or induced experimentally (e.g., by mirror in amputees), the sensation that a limb exists, even when it is completely absent, may indicate the imprinting and awareness of that body zone established prior to the injury or even in the innate body map [58]. However, in healthy individuals, it is remarkable that the brain can so quickly and easily be fooled during the RHI into accepting a different hand as being part of one's own body, especially as the physical body is intact and the body shape remains integral. Given the innate and permanent experience of having a physical body, the combined data on healthy subjects and individuals with body deafferentation suggest that although the mere sight of one's physical body seems to predominate in triggering the immediate perception of the corporeal experience, the brain's construction of the body might rely on multiple perceptions and the temporal constancy of offline body representations in the human brain. So, if the sight of the physical body does not seem to be critical, are distortions and misperceptions of the body the result of integrated changes in the neural representation of these body parts? Moreover, what are the neural changes that may trigger the unity assumption of body awareness?

## 4. Sensory Motor Experiences in Body Recalibration

There is increasing indirect evidence of changes in somatosensory and motor body maps and their interactions in response to changing afferent inputs and efferent outputs. After an SCI, for example, peripheral body alterations can modify the central representation, as large areas of the somatosensory cortex are deafferented, while areas within the motor cortex linking to the descending motor system are deafferented.

Most of the reorganization of cortical body representation following SCI is documented by the use of techniques such as transcranial magnetic stimulation (TMS) and functional magnetic resonance imaging (fMRI). While TMS allows researchers to observe excitability changes and map cortical zones in the preserved and deafferented areas, fMRI is useful for viewing variations in cortical activity in areas both close to and distant from these regions. Separately, or in combination, these techniques provide indirect evidence of body cortical reorganization and provide an excellent opportunity to test the sensory motor hypothesis concerning the modified neural representation of the body.

Several seminal studies have documented how single-pulse TMS delivered to the primary motor cortex of patients with a cervical SCI induces an increase in the motor evoked potentials (MEPs) recorded in two unaffected muscles proximal to the spinal injury, immediately above the lesion [59]. In addition, when delivered at rest, focal stimulation evoked MEPs in preserved muscles at shorter latencies. More sophisticated experimental combinations providing information on the influence of different cortical regions contingent with the deafferented areas have been documented in patients with thoracic lesions. These show enlargement of the motor cortical territories controlling intact body parts, larger MEP amplitudes, and shortened latencies in the responses of unaffected proximal muscles [60]. These data clearly suggest that the motor cortex and/or its corticospinal projection system may have undergone “reorganization.” Interestingly, this effect was observed even when stimulating muscles that were distant from those directly affected by the lesion, supporting the view that patients with SCI experience widespread postlesional alterations to brain regions that represent, construct, and control the body. Against this, studies of paraplegics have reported reorganization for an arm muscle represented near the deafferented area, but not for another arm muscle that is more distant from this area [61]. It follows that although body areas close to the lesion appear to undergo neural reorganization, changes to distant areas are less predictable. Understanding and quantifying the impact of cortical reorganization on brain areas close to and distant to injury, along with rehabilitative strategies, remain controversial and a fundamental topic for future research.

How cortical adaptation to local changes in the body reflects the integration of cortical plasticity and cortical reorganization is best investigated using fMRI. Lotze et al. [62] compared cortical activity during executed movements of the right elbow, right thumb, lip, and right foot and imagined movements of the right foot in healthy subjects and patients with a complete or partial SCI. During lip and thumb movements, the BOLD signals of both patient groups were not different from those of the control group. In patients with a complete SCI, the BOLD signal for the elbow movements was significantly displaced towards the disconnected M1 (primary motor cortex), with a shift of 13.3 mm. This displacement was not observed in the patients with an incomplete SCI. Imagined and executed movements of the foot did not produce a shift or elicit a significant M1 activation in the patients with a complete SCI. The observed displacement of the elbow representation in the patients with a complete SCI was interpreted as evidence of primary motor reorganization, as this body territory is proximal to the disconnected cortical area. In the patients with cervical lesions, evidence of adaptation in the motor cortex indicated a clear effect of the lesion level. Unlike patients with paraplegia [63], the shift in representation in cases of cervical lesions involved the M1 region that controls tongue movements; this area was displaced medially and superiorly, with a shift of 12.8 mm towards the neighbouring disconnected hand cortex. Moreover, the shift correlated with the SCI level and the amount of spared motor function; greater hand-tongue separation was linked to greater movement impairment and a higher injury level. However, pathological changes following SCI are not confined to the corticospinal tract but also extend to sensory pathways and the primary somatosensory cortex (S1) [64]. Shifts have been documented in S1 during mouth, thumb, little finger, and big toe stroking. The thumb and little finger activations were displaced by 7 and 13 mm, respectively, towards the midline. The greater medial shift in little finger activation coincided with lower fractional anisotropy in S1 hand area, as measured by diffusion tensor imaging (DTI), which would normally innervate the legs of patients with paraplegia [64].

Collectively, these studies support the presence of neural plasticity, with the greater enlargement of cortical representation sites in regions that are nearer to, rather than more distant from, the deafferented site. They also support the plasticity interpretation of the observed shifts in cortical motor and somatosensory representations of specific body parts. Finally, reactive changes have also been found to occur in spontaneous neuronal activity of radically affected areas and slightly affected fringes adjacent to intact cerebral maps. As a consequence, these patients undergo substantial plastic changes in the neural representation of the body, which are associated with alterations in the cortical representation sites, cortical excitability, and brain connectivity.

The neural changes in these different areas destabilize the body representation and would be expected to produce experiences of profound alteration. However, conscious feeling and the conception of one's own body as an integrated whole are in fact minimally disturbed. The different topographic organization is nevertheless fundamental for the functional proprieties of the body but the neural modifications do not reach conscious awareness of body image, suggesting we should consider not only the sensory and motor loss but also the experience as potential drivers of changes in body awareness. Additionally, SCI patients are not entirely “disembodied” but instead have an altered or transformed awareness of their body. Although no longer able to move or feel, they do experience a need to attend to bodily care, becoming vigilant in this regard, often demonstrating heightened body attention [65, 66]. Maintaining a precise visual body representation following deafferentation could play an important role in preventing maladaptive neural plasticity, as there is evidence that referred sensations, presumably linked to cortical remapping, can preserve precise topographic organization following SCI. For example, the increased excitability observed in individuals with SCI could underlie the reorganization of cortical pathways in a maladaptive way, affecting the neuropathic pain that some of these patients experience, although this correlation is yet to be verified [67]. Accordingly, the extent of the body image distortion involves the plastic modification of the cortical representation of the body in key areas for corporeal awareness and pain perception [68].

Therapeutic approaches with repetitive TMS could be used to reduce neuropathic pain, as well as somatosensory and motor impairment and spasticity, in patients with complete and incomplete SCI [69]. The repetitive stimulation would modulate excitability and promote cortical and subcortical reorganization, helping to restore functionality. The therapeutic use of these techniques exploits the momentary plasticity that follows the establishment of a deafferented area until clinical recovery causes a decrease of reorganization and a shift towards normalized measures [61]. The high malleability of the neural connections defining the brain's precise topography suggests that rehabilitation might exploit the potential for residual central organization within the cortex in order to preserve the body representation and reduce its alterations.

## 5. Synthesis and Conclusion

The difficulty of assigning precedence to any one of the three views examined here serves to confirm that the distortions and mismatching of body representation involve the synthesis of multiple sources of information. Findings related to plasticity and awareness indicate the existence of several body maps in the same individual, and these maps may be said to shift, distort, disappear, and expand. In some cases, the possibility of major changes resulting from multisensory information flows and the integration of neural activity leads to labile changes in the perceived size of body parts and other unconscious distortions of body configurations, which are matched or mismatched to the somatotopic map and painful sensations. Indeed, plastic changes in the physical body, the illusory constructed body, and the phantom body mean that curious clinical phenomena and prosthetics—though unlikely and even conflicting—can coexist in unique ways, generating temporary, dynamic, and manipulable corporeal awareness.

Neural plasticity can tell us much about therapeutic approaches to changing how the distorted body is perceived and felt. A deeper understanding of the different and potentially conflicting sources informing mental body maps may facilitate the development of effective treatments for bodily discrepancies or distortions in altered functions. For example, procedures that update hard-wired, offline bodily representations may (at least temporarily) suppress the pathological phenomena providing the appropriate input. Therapies designed to preserve or restore precise cortical topography (even when sensations are transferred to a different cortical territory) may prevent maladaptive plasticity, preserving body representation and potentially mitigating nociceptive and somatic abnormalities. Even in these apparently abnormal situations, a sense of normality can be temporarily restored, inviting explorations of how systematic rehabilitation might achieve similar outcomes.
